# Functional recovery after implantation of artificial nerve grafts in the rat- a systematic review

**DOI:** 10.1186/1749-7221-4-19

**Published:** 2009-10-25

**Authors:** Nektarios Sinis, Armin Kraus, Nikolaos Tselis, Max Haerle, Frank Werdin, Hans-Eberhard Schaller

**Affiliations:** 1 Clinic for Hand, Plastic, Reconstructive and Burn Surgery, BG Trauma Center, Eberhard-Karls University, Schnarrenbergstrasse 95, D-72076 Tuebingen, Germany; 2 Dept of Hand and Plastic Surgery, Orthopaedic Hospital Markgroeningen, Kurt-Lindemann-Weg 10 D-71706 Markgroeningen, Germany; 3 Dept of Radiotherapy, Hospital of Offenbach, Starkenburgring 66, D-63069 Offenbach, Germany

## Abstract

**Purpose:**

The aim of this study was to compare functional data of different nerve-gap bridging materials evaluated in rat experiments by means of a systematic review.

**Materials and methods:**

A systematic review was conducted, searching MEDLINE, HTS and CENTRAL to identify all trials evaluating functional recovery of artificial nerve conduits in the rat model.

**Results:**

There was a trend towards a favourable outcome of conduits coated with Schwann-cells compared to the plain synthetics. Histomorphometry, electrophysiology and muscle-weight correlated poorly with functional outcome.

**Conclusion:**

Schwann-cell coated conduits showed promising results concerning functional recovery. Further standardization in outcome reporting is encouraged.

## Introduction

Peripheral nerve injury is common in trauma patients, since, 4.5% of all soft-tissue injuries are accompanied by defects of peripheral nerves [1]. The first attempts in repairing peripheral nerve injuries were made in the 17^th ^century [2]. In the 19^th ^century, various options for the surgical management of peripheral nerve injuries were under debate, such as stretching the nerve, mobilizing the nerve by joint flexion or bone shortening or bridging the defect with various organic or synthetic materials [3]. In the late 20^th ^century, it became clear that tension across a nerve repair site negatively affects regeneration which led to preference of nerve grafting over manipulating procedures [4]. Despite the well-known benefits of nerve grafting, donor site morbidity must be taken into consideration. To solve this problem, artificial nerve conduits are in evaluation, mainly in animal models. However, the most cost sparing animal model to start with remains the rat. Herein, different nerves (i.e. median nerve, sciatic nerve, facial nerve, etc.) were used in the past to demonstrate the efficacy of numerous materials and concepts (resorbable vs. non-resorbable, cellular vs. acellular, etc.). This plenty of different experimental studies in the rat were conducted with different techniques, materials, and aims making a direct comparison of the data very difficult. The aim of this study was to quantitatively compare these different materials applied in the rat in order to find the best concept for tubulization in the rat peripheral nerve.

## Materials and methods

### Inclusion criteria

Since various parameters were analyzed in the different studies, the overall aim was defined to compare functional criteria among different concepts. Therefore, the publications reviewed for this article all refer to the functional efficacy of artificial nerve guidance tubes in the rat model. This systematic review includes meta-analyses and overview articles published between the years 2000 and 2008. Furthermore, controlled experimental studies were included. Studies were rated as "controlled" if a comparison group existed. Studies were only included if they evaluated at least one artificial material (e.g. studies testing vein grafts were excluded). Further, the material should be applied in the upper or lower extremity of the rat (rat facial nerve models were excluded since there are only of limited clinical worth - see discussion). Finally, only those studies were utilized where functional analysis was performed (e.g. gait analysis or grasping tests).

### Exclusion criteria

Uncontrolled studies as well as case reports, description of the surgical methods, in vitro studies, studies performed on animals other than the rat in order to warrant comparability were not analyzed.

### Search strategy

For literature search, the following databases were used:

#### Medline database

The Cochrane Central Register of Controlled Trials (CENTRAL)

Health Technology Assessment Database (HTS)

Pub-med "related articles" function for included studies

The following key words were used: "nerve", "rat", "conduit", "tube", "regeneration", "artificial". Numerous combinations of these terms were individually applied and the results compared using Medline's search history feature. Articles were also identified by using the function "related articles" in PubMed).

### Management of references

The bibliographic details of all retrieved articles were stored in an Endnote file. We removed duplicate records resulting from the various database searches. The sources of identified articles were recorded in a "user defined field" of the Endnote file.

### Study selection

Two members of the review team independently assessed the titles and abstracts of all identified citations. English or German language was a restriction. Decisions of the two reviewers were recorded (order or reject) in the Endnote-file and then compared. Any disagreements were considered by a third reviewer.

Two reviewers evaluated the full text of all potentially eligible papers and made a decision whether to include or exclude each study according to the inclusion and exclusion criteria specified above. Final decisions on papers were then recorded in the Endnote file. All studies that did not fulfil all of the criteria were excluded and their bibliographic details listed, with the reason for exclusion.

### Data extraction strategy

Two reviewers independently recorded details about study design, interventions, patients and outcome measures in a predefined Windows Excel form. A small sample of studies with high likelihood for inclusion and exclusion served to pretest the data forms. A third reviewer resolved any discrepancies if the two reviewers disagreed. Bibliographic details such as author, journal, year of publication and language, were also registered. If any data were not indicated in the text but shown in figures or graphs, data were estimated therefrom.

### Statistics

Thorough consultation with a statistician (Department of Medical Biometry, University of Tuebingen) supplied evidence that direct statistic comparison of the included studies was not possible due to a variety in measured parameters and timepoints too large to subsume them in statistic tests. We therefore had to constrain our work to a descriptive approach.

## Results

Entering the above mentioned keywords yielded 384 references in all scanned databases, From these, 62 were selected for full-text assessment according to the applied criteria. The selection strategies reduced the references to a number of 12 publications that were compared in this article. [see additional files 1 and 2].

### Functional assessments

Ten of 12 studies used the sciatic functional index (SFI) as a parameter for functional limb recovery, 1 study by Piquillod used the peroneal score [5], the study of our group performed on median nerve utilized a grasp test [6]. However, the SFI remains the most popular functional test among experimental surgeons on that field. This test is described in detail elsewhere [7]. Comparing the classic silicone tube to other materials such as glutaraldehyde-crosslinked gelatine or poly(l-lactic acid), PLLA showed better results [8,9]. Chen MH could show significantly better SFI (-38,1 vs -53,2) for glutaraldehyde-crosslinked gelatine compared to empty silicone tubes 24 weeks after implantation in their study published in 2006. Evans reveal an SFI of 74,4 for empty silicone compared to 83,7 for PLLA in bridging a gap of 12 mm, measured after 16 weeks, this result being not significant.

In the results of Pilliquod [5] utilizing the peroneal score [10], there was no significant difference between nerve suture causing no gap, and a collagen tube with coating layers of poly(lactide-co-glycolide) releasing either no or various concentrations (6 ng/d, 10 ng/d or 15 ng/d) of glial cell line-derived neurotrophic factor (GDNF) after 12 weeks across a distance of 3 mm. Peroneal score was 1152 for simple suture, 1161 for PLGA tube without GDNF. For the factor-emitting tubes, peroneal score was 1176, 1233 and 1203 for 6 ng/d, 10 ng/d and 15 ng/d of GDNF emission. Furthermore, Schwann-cell alignment in the conduit (either 2-dimensional or 3-dimensional) was shown to have an influence on SFI regeneration. According to a study of Kim published in 2007 [11], 3-dimensionally aligned Schwann cells lead to a better SFI than 2-dimensionally aligned Schwann cells (-60, Vs -87) over a gap of 10 mm measured after 12 weeks. Significance level was not revealed due to the low case number (n = 6 per group). According to a study by Mohammad conduits manufactured of human amnion were not superior to autologous nerve grafts across a distance of 10 mm concering SFI, but almost equalled nerve grafts in this point 16 weeks after implantation (SFI -93,7 vs SFI -92,9) [12]. It surprises in these results, however, that also empty silicone tubes showed SFI regeneration only little inferior to amnion and nerve grafts (-91,0). In contrast the above-mentioned study of Evans [9] shows inferior results of an empty silicone tube compared to autologous nerve grafts (SFI 74,4 vs 86,9), observed 16 weeks after implantation over a gap of 12 mm. In another study by the group of Evans from 2000 investigating late results after conduit implantation over a 10 mm gap, an empty PLLA conduit in comparison to an autologous nerve graft showed slightly lower SFI values (105 vs 113 m, not significant) 32 weeks post implantation in the sciatic nerve of 31 Sprague-Dawley rats [13]. Rutkwoski investigated the capabilities to bridge a 10 mm nerve defect of a poly(D, L-lactic acid) (PDLLA) tube either with or without micropatterned lumen and with or without Schwann cell seeding respectively [14]. They found significantly earlier onset of functional recovery and higher peak recovery for the experimental group where Schwann cell seeded tubes with a micropatterned lumen were utilized.

In none of the included studies however, SFI reached with an artificial conduit essentially surpassed SFI achieved after nerve grafting or suture. Chen CJ et al. found a significantly improved SFI for a 15 mm nerve defect by utilizing bone marrow stroma cell coated silicone tubes compared to empty silicone tubes after 10 weeks [15]

### Histomorphometric analysis

In the respective studies, a various number of histomorphometric parameters are reported such as total axon number, number of myelinated fibres, total nerve area or myelin thickness. High myelin thickness correlates well with high sciatic functional index in the studies of Nie. from 2007 [16], where autografts, empty PLGA-conduits and PLGA-conduits with ectomesenchymal stem cells are compared. After 16 weeks, the investigators found higher SFI in the experimental groups with higher myelin thickness (autograft and PLGA conduits containing stem cells compared to empty PLGA tubes). Likewise, they showed positive correlation of large nerve area and large fibre density to higher SFI values (autografts and stem-cell containing conduits possessed larger nerve areas and higher fibre density than empty PLGA conduits). High axon number was directly correlated with good functional recovery in the work of Chen CJ et al [15]. According to results of our group, high myelin thickness also lead to better results in the grasp test than low myelin thickness 36 weeks after surgery [6]. In contrast, our group achieved contrary results when fibre density and the number of myelinated fibres was compared to the functional index, as in the experimental group with the lowest number of myelinated fibres and the lowest fibre density per mm, functional results were best [6]. The fact that functional performance was better for experimental groups with lower nerve fibre density is also seen in other studies. The group of Tomita [17] could show higher fibre density in all experimental groups undergoing surgery (whole nerve graft, fascicular nerve graft and whole nerve graft and silicone tube) compared to the control group undergoing no surgery and serving as the gold standard for functional performance. Also in the two above mentioned studies from Evans form 2000 and 2002, functional outcome is better for the experimental groups showing low fibre density than in the groups with high fibre density [9,13]. In the study of Rutkowski from 2004 mentioned above [14], there was no difference in nerve area and axon count for the functionally superior group (micropatterned PDLLA tube seeded with Schwann cells) in comparison to the control groups (micropatterned PDLLA tube unseeded, unpatterned PDLLA unseeded and unpatterend PDLLA seeded).

### Muscle weight

Several of the studies used weight analysis of a muscle innervated by the operated nerve as a parameter for regeneration, such as the studies of Tomita, our group and Evans [6,9,13,17]. Results however are mostly contradictory.

In their study of 2002, the group of Evans found higher SFI values in the experimental group with higher weight of the gastrocnemius muscle [13], as well as the group arond Chen CJ et al. comparing BMSCs coated silicone tubes to empty silicone tubes [15]. Our group could only partially confirm these results [6]. Later than 32 weeks after surgery, functional performance was almost equal in the control group, in the autograft group and in the Schwann cell seeded tube group. Muscle weight in the experimental groups however was only 71.8% and 67.1% of that of the control group. On the other hand, it was not astonishing that the group showing no functional regeneration (empty TMC/CL conduit) had the lowest muscle weights measured (14% of the control group weight). Remarkably, in the results of Evans from 2002, the experimental group showing the best functional outcome (PLLA conduit filled with Schwann cells in a concentration of 1 million cells per ml) had the second lowest muscle weight.

### Electrophysiology

For electrophysiological tests, the respective studies utilized either compound muscle action potential (CMAP) or nerve conduction velocity. Tomita [17] found highest CMAP for their control group undergoing no surgery. They additionally found almost equal CMAP (68,1% and 73,2%) of the sciatic nerve 12 weeks after surgery in the functionally equal study groups (fascicular nerve graft, nerve graft and silicone tube, SFI -50 in both cases). Chen MH et al. [8], comparing glutaraldehyde crosslinking gelatin conduits with silicone tubes, found higher CMAP (0,80 mV) in the glutaraldehyde crosslinking gelatin conduits group which showed higher SFI (-38,1), whereas they found lower CMAP (0,46 mV) in the silicone tube group which showed lower SFI (-53,2) after 24 weeks. Chen CJ et al. found higher CMAP in their experimental group of BMSC coated silicone tubes than in their control group of empty silicone tubes as well after 10 weeks. In the study of our group [6], nerve conduction velocity was measured in the control group, autograft group and TMC/CL with Schwann cell tube group after 36 weeks. In all 3 groups nerve conduction velocity was equal (35 m/s), which was in accordance to equal results in the functional grasp test.

## Discussion

With peripheral nerve injury being a common and serious problem [2] there is a demand for surgical repair. Due to the presence of donor site morbidity after harvesting of nerves and the results that remain still far from satisfactory after nerve grafting operations the hope of improving the results by using artificial nerve conduits, lead to an intensive research on that field [18-21]. Most of these studies are performed in the rat, although we know about this species to have excellent regeneration capabilities after peripheral nerve injury [7]. However, the rat is still an attractive animal model since the costs of these animals are low and the rats are easy to handle. Therefore, we limited our literature search to this kind of studies. Furthermore, functional recovery appeared to be the fundamental outcome parameter to us, therefore we included only studies which quoted any of such tests. Rat models of facial nerve tubulization were not included because this reconstructive modality is of minor importance in trauma surgery of peripheral nerves which mostly includes the upper extremity.

During the 20^th ^century, a variety of non-biological materials such as cellulose esters, gelatine tubes, rubber and plastics were under experimental evaluation [22]. Particularly, Dahlin and Lundborg [23] showed that in human short nerve defects can successfully be treated with silicone tubes, with best results in large proximal nerves such as the median and ulnar nerve [24]. In addition to non-absorbable tubes, bioabsorbable tubes were tested experimentally and also under clinical conditions [25-29]. As a further development, tissue engineered tubes enriched with elements such as specific cells or neurotrophic factors were presented. Especially Schwann-cell coated non-biological conduits showed promising results, as shown in the studies of Evans, our own results, the group of Rutkwoski and in the work of Gravvanis [6,9,14,30,31]. Neurotrophic factors used in bioengineered tubes were nerve growth factor (NGF) [32], brain derived neurotrophic factor (BDNF) [33] or glial derived nerve growth factor (GDNF) [5,34]. In the studies reviewed for this article, there was a tendency towards better regeneration with cellular filled conduits compared to plain acellular tubes.

Judging the comparability of the utilized materials was a challenge in this review, since the studies included were characterized by a large variety of measurement timepoints and outcome parameters quoted. According to statistical consultation, this fact made any statistical comparison of the various studies unfeasible. We encourage any efforts to standardize outcome measurement in the field of nerve tubulization to alleviate further reviews. Yannas and Hill suggested a method for comparing regenerative capabilities of various tubulisation materials with different gap lengths being chosen in different animal models [35]. They used normalized length of bridged nerve gaps in various species and determined the point of 50% successful myelinisation of the fitted fibers for various materials. The authors found poly(lactic acid), a copolymer of lactic acid and ε-caprolactone and a natural polymer of type I collagen to induce a significantly better axon outgrowth than ethylene-vinyl acetate copolymer tubes. Furthermore, they found fibroblast growth factor (FGF) to enhance myelinisation, but nerve growth factor (NGF) did not, nor did addition of extracellular matrix molecules such as laminin oder fibronectin. Concerning non-cell-coated materials, the group of Waitayawinyu et al. [36] found a type I collagen tube to produce superior results compared to polyglycolic acid tubes (PGA) concerning muscle contraction forces, axon counts and weight of the innervated muscle. Interestingly, the group of Clavijo-Alavrez et al. [37] found not difference neither in SFI, gastrocnemius weight nor myelinated nerve area when they compared polycaprolactone nerve guides, polycaprolactone/collagenous beads composite guides and polyglycolic acid guides in elder (11 months old) Sprague-Dawley rats. Obviously, sharply decreased regenerative potential of peripheral nerves cannot be enhanced by any artificial material.

Muscle weight measured as a possible outcome parameter also correlated only partially with functional indices, indicating that muscle hypertrophy is not a suitable characteristic of muscle strength or function, regarding the results of our group [6] or those of the group of Evans form 2002 [9]. It would be worthwhile to find a histomorphometric or electrophysiological parameter that better correlate with functional indices to facilitate transmission of in vitro data to in vivo experiments. However, it was interesting to observe that all histomorphometric or electrophysiologic measurements correlated only poorly with functional outcome in the studies that were reviewed for this work. With respect to any future clinical implementation, focus should be fixed on functional rather than technical parameters.

Regarding functional outcome, motor and sensory function tests can be distinguished as cited in the review by Vlegeert-Lankamp [38]. Concerning sensory function tests, animal reflexes after electrical stimulation of the hindlimb have been measured, the operated nerve has been pinched by a pair of forceps distal or proximal to the graft location and muscle contraction or leg retraction was measured, or the footpad was pinched and the withdrawal response was measured. As sensory answer is another parameter indicating whether a nerve has regenerated through an artificial conduit, we recommend to take it into account assessing the degree of recovery, even if there is the well-known preference of motor over sensory rehabilitation. For measuring muscle tetanic force, the muscle innervated by the sciatic nerve is cut, the tendon is fixed to a force transducer and the nerve is stimulated measuring the maximal force. Despite valuable information about muscle force, the applicability of the test is limited to a single experiment, making observations over a time course unfeasible. Walking track analysis is a frequently used method to evaluated peripheral nerve regeneration as it provides information about both nerve motor and sensory function and muscle force. Among various walking track tests, the SFI is still in widespread use, as it is in the articles matching our inclusion criteria, although it can be regarded as outdated in some aspects. The SFI is calculated by a formula using printlength, hindpaw toe spread (distance from first to fifth toe) and intermediate toe spread (second to fourth toe). As contractures of the operated hindlimb may occur over the time, the index is susceptible for errors in long-term measurements. More advanced methods of motion analysis such as those described by Bozkurt. [39] or by Meek [40] utilizing video analysis and taking both dynamic and static gait parameters into account are promising. As already proposed by Geuna [41], we recommend to standardize a combination of advanced motor and sensory tests to make future results from research in the field of peripheral nerve regeneration more comparable. Further studies will have to show the best motor and sensory functional tests together with their ideal combination.

## Conclusion

Among the artificial nerve conduits analysed for this review, especially those coated with Schwann-cells showed promising results concerning functional recovery. Functional recovery only partially correlated with histomorphometric parameters. Due to various different outcome parameters in common use, comparability of the studies is very limited. We encourage any standardization in this field of research utilizing both advanced motor and sensory functional tests to facilitate further meta-analyses.

## Abbreviations

SFI: sciatic functional index; PLLA: poly(l-lactic acid); PLGA: poly(lactide-coglycolide); TMC/CL: trimethylenecarbonate-co-epsilon-caprolactone; PDLLA: poly(D, L-lactide); CMAP: compound motor action potential.

## Competing interests

The authors declare that they have no competing interests.

## Authors' information

NS is a senior surgeon in plastic and reconstructive surgery. His scientific work has mainly been concerned with bioartificial nerve conduits in the rat model.

## Authors' contributions

NS is responsible for the study design of this review and the writing of the manuscript. AK did the literature search and co-writing of the manuscript. NT and MH worked on reviewed the existing literature and prepared them for inclusion or rejected them. FW worked on data processing and tabulation. HS edited the text of the manuscript and consulted in study design. All authors read and approved the final manuscript.

## Supplementary Material

Additional file 1 **studies utilizing artificial nerve grafts in the rat**. overview of the various studies utilizing artificial nerve grafts in the rat and evaluating functional outcome. Click here for file

Additional file 2 **studies utilizing artificial nerve grafts in the rat**. overview of the various studies utilizing artificial nerve grafts in the rat and evaluating functional outcome. Click here for file
