# Supplementary figures and images for: Functional recovery after implantation of artificial nerve grafts in the rat- a systematic review
Source: J Brachial Plex Peripher Nerve Inj. 2009 Oct 25;4:19. doi: 10.1186/1749-7221-4-19 (PMC2770034; doi:10.1186/1749-7221-4-19)

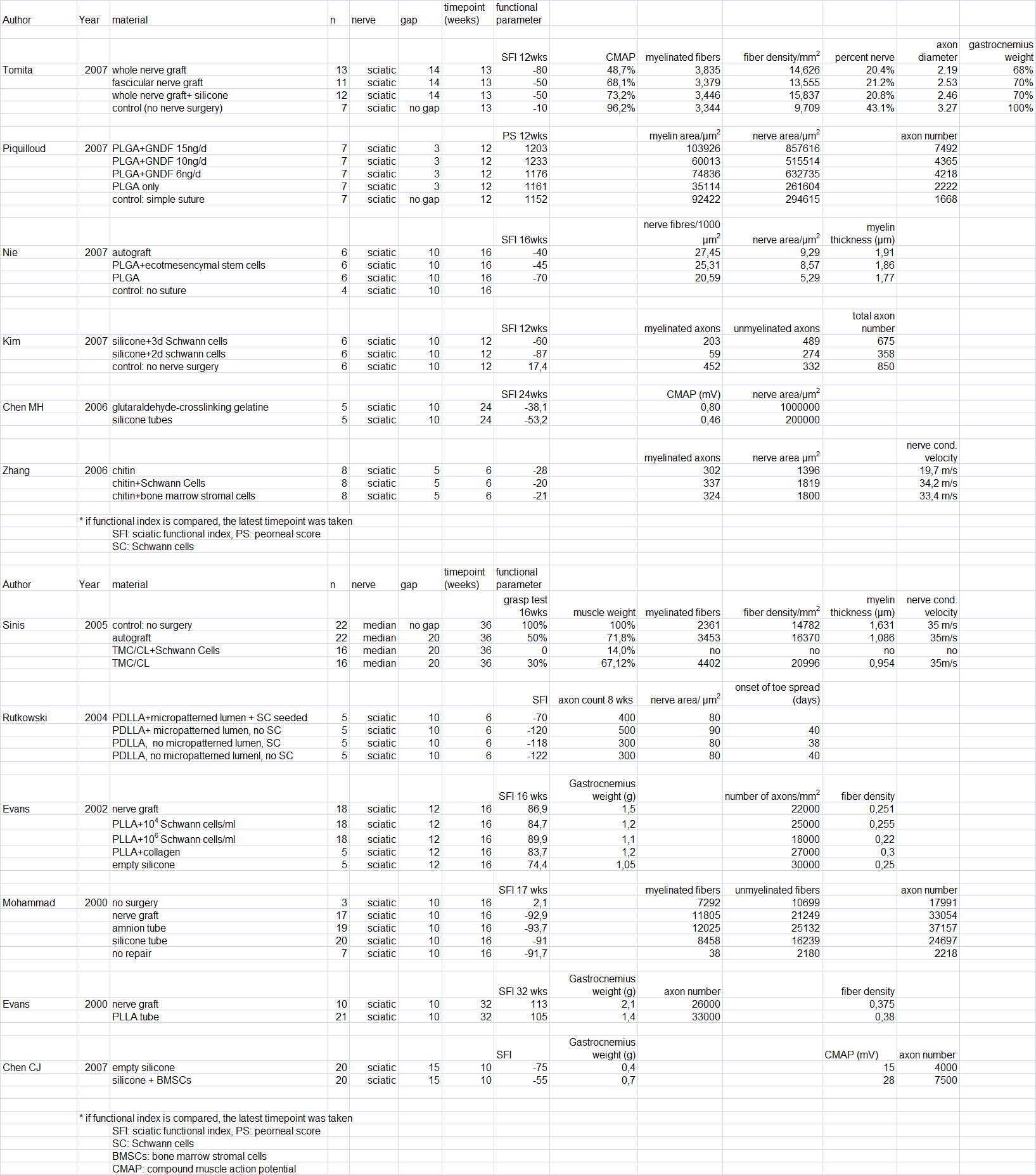

Supplement: Additional file 1 — studies utilizing artificial nerve grafts in the rat. overview of the various studies utilizing artificial nerve grafts in the rat and evaluating functional outcome. [file 1749-7221-4-19-S1.TIFF]

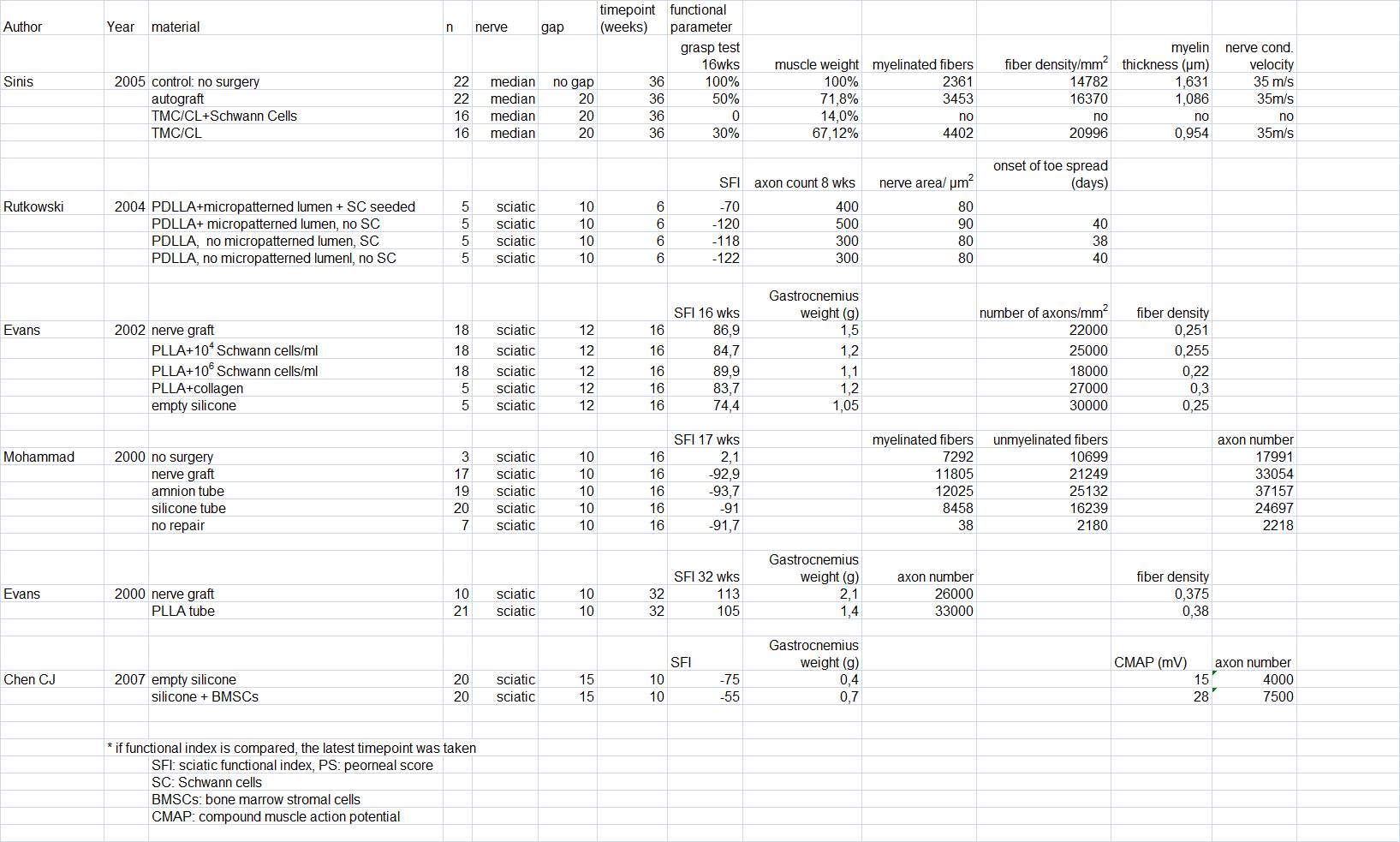

Supplement: Additional file 2 — studies utilizing artificial nerve grafts in the rat. overview of the various studies utilizing artificial nerve grafts in the rat and evaluating functional outcome. [file 1749-7221-4-19-S2.TIFF]
